# Aortic valve repair with sinus plication for a regurgitant bicuspid aortic valve: a case report

**DOI:** 10.1186/s44215-024-00184-0

**Published:** 2025-01-14

**Authors:** Atsutaka Aratame, Takashi Kunihara, Toshio Baba, Masanori Sakaguchi, Yosuke Sumii, Mikado Fukuda, Yosuke Takahashi

**Affiliations:** 1 Bell-Land General Hospital, 500-3, Higashiyama, Naka-ku, Sakai city, Osaka 599-8247 Japan; 2 Department of Cardiac Surgery, The Jikei University School of Medicine, 3-25-8 Nishishinbashi, Minato-ku, Tokyo, 105-8461 Japan; 3 Osaka Metropolitan University Graduate School of Medicine, 1-4-3, Asahimachi, Abeno-Ku, Osaka, 545-8585 Japan; 4 Bell-Land General Hospital Department of Cardiovascular Surgery, Sakai city, Osaka 599-8247 Japan

**Keywords:** Aortic valve repair, Sinus plication

## Abstract

**Background:**

Repair of the regurgitant bicuspid aortic valve is an attractive alternative to valve replacement. Although good long-term outcomes have been reported, postoperative aortic stenosis remains a major late cause of repair failure in bicuspid aortic valves. Sinus plication is effective for creating a more symmetrical commissural angle, leading to a decrease in the mean transvalvular pressure gradient. Herein, we report a successful case of aortic valve repair with sinus plication for a regurgitant bicuspid aortic valve.

**Case presentation:**

The patient was a 34-year-old man with bicuspid aortic valve, severe aortic regurgitation, and left ventricular dilatation. Echocardiography revealed an eccentric aortic regurgitation jet caused by a fused cusp prolapse. The commissural angle was approximately 136°, classified as asymmetrical. The patient underwent surgery via median sternotomy. Cardiopulmonary bypass was initiated using the ascending aortic and single two-stage atrial cannulation with left ventricular venting via the right superior pulmonary vein. After the ascending aorta was cross-clamped, transection was performed 5–10 mm above the sinotubular junction. Based on the valve configuration, annuloplasty, sinus plication, and ascending aortic replacement were planned in addition to triangular resection of the bulking raphe tissue with central cusp plication. Mattress sutures of 4–0 polypropylene, reinforced with autologous pericardium, were placed outside the fused cusp for sinus plication. The sutures extended from the basal ring to the sinotubular junction, reducing the sinus circumference by approximately 15 mm. Intraoperative transesophageal echocardiography revealed trivial aortic regurgitation and modified commissural angulation. The operation, cardiopulmonary bypass, and aortic cross-clamping times were 311, 129, and 95 min, respectively.

The clinical course was uneventful, without major complications. Postoperative echocardiography demonstrated an improved commissural angle. Follow-up echocardiography demonstrated trivial aortic regurgitation and a decreased mean transvalvular pressure gradient.

**Conclusions:**

Sinus plication may be one of the most effective repair techniques for asymmetrical bicuspid aortic valves, especially in cases without Valsalva sinus dilation. This technique helps avoid postoperative aortic valve stenosis.

## Background

Bicuspid aortic valve (BAV) is the most common congenital cardiovascular anomaly. It features variable degrees of fusion between two cusps and the presence of a non-fused cusp. Some individuals with BAV require treatment at a young age owing to aortic regurgitation (AR) or aortic dilatation [1]. Aortic valve replacement is the standard treatment for AR; however, it is associated with valve-related complications such as reoperation, stroke, and bleeding [2]. Mechanical valves require lifelong anticoagulation, which carries risks. Repair of the regurgitant bicuspid valve has emerged as an attractive alternative to valve replacement. Although good long-term outcomes have been reported, postoperative aortic stenosis and residual AR remain major causes of repair failure in BAV [3–5].

To avoid postoperative aortic stenosis, Schneider et al. advocated plication of the fused sinuses of Valsalva to address commissural angle asymmetry (sinus plication), leading to better durability of BAV repair [6]. Arimura et al. demonstrated that sinus plication in the BAV model decreased the mean transvalvular pressure gradient and effectively controlled AR in an experimental pulsatile flow simulator [7]. Based on these positive results, we performed aortic valve repair, including sinus plication, for a patient with asymmetrical BAV and severe AR.

## Case presentation

**Fig. 1:**
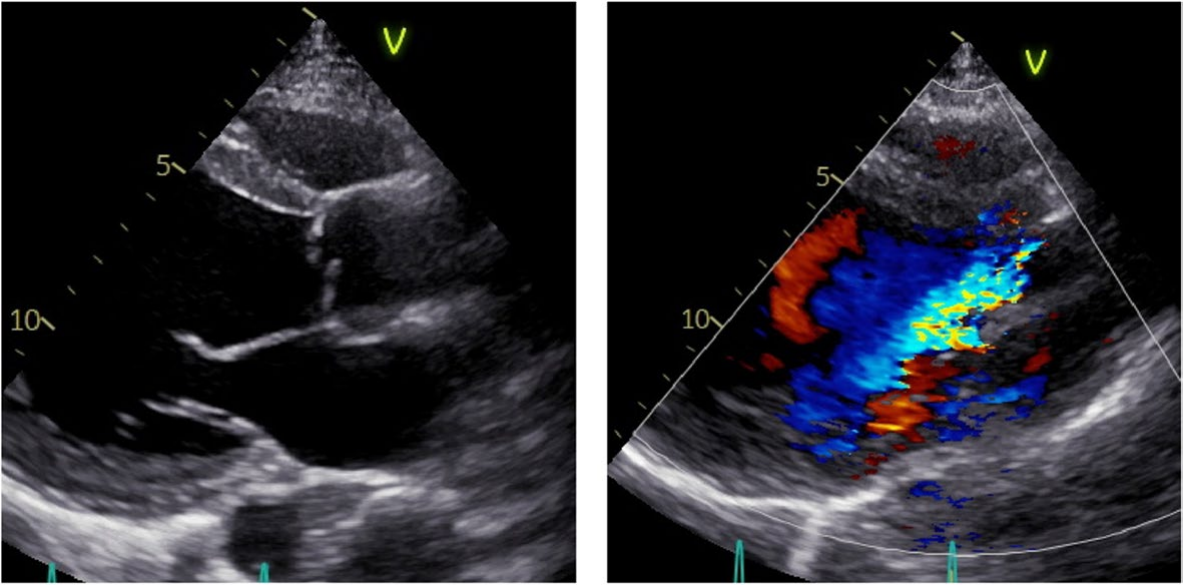
Preoperative echocardiography showing the prolapsed fused cusp, eccentric jet, and severe aortic regurgitation

**Fig. 2:**
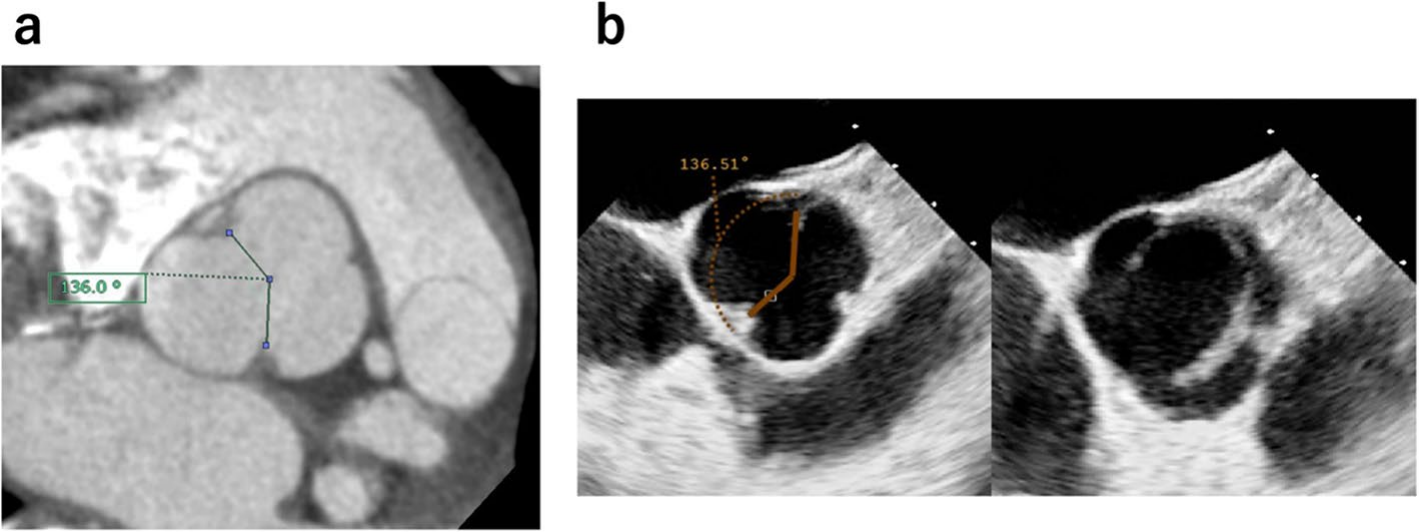
Preoperative contrast-enhanced computed tomography and transesophageal echocardiography showing the commissural angle of approximately 136°. The commissural angle was measured by using transesophageal echocardiography on short-axis view. Each line is drawn from the center of cusp coaptation to the two commissures

The patient was a 34-year-old man (body surface area: 1.86 m^2^) diagnosed with BAV, severe AR, and left ventricular dilatation. Echocardiography revealed an eccentric AR jet caused by a fused cusp prolapse (Fig. 1), with a mean transvalvular pressure gradient of 18.0 mmHg. Contrast-enhanced computed tomography revealed an ascending aortic diameter of 42.3 mm, with aortic root dimensions of 31.3 mm at the aortic annulus, 35.9 mm at the sinus of Valsalva, and 26.0 mm at the sinotubular junction (STJ). The commissural angle was approximately 136°, and the fusion leaflet of the left and right cusps was classified as very asymmetrical (Fig. 2) [8]. The patient underwent surgery via median sternotomy. Cardiopulmonary bypass was initiated using the ascending aorta and single two-stage atrial cannulation. A cannula was inserted through the right superior pulmonary vein to vent the left ventricle. The ascending aorta was cross-clamped, and transection 5–10 mm above the STJ was performed. Blood cardioplegia was administered regularly into the coronary ostia. The geometric heights of the right, left, and noncoronary cusps were 18, 20, and 22 mm, respectively. The effective height of the non-fused cusp was 10 mm. The free margin length of the non-fused cusp was 34 mm and 45 mm for the fused cusp. The aortoventricular diameter (AVD) could not be precisely evaluated because the 24-mm Hegar dilator could not pass through the level of leaflets due to the fused cusp.

**Fig. 3:**
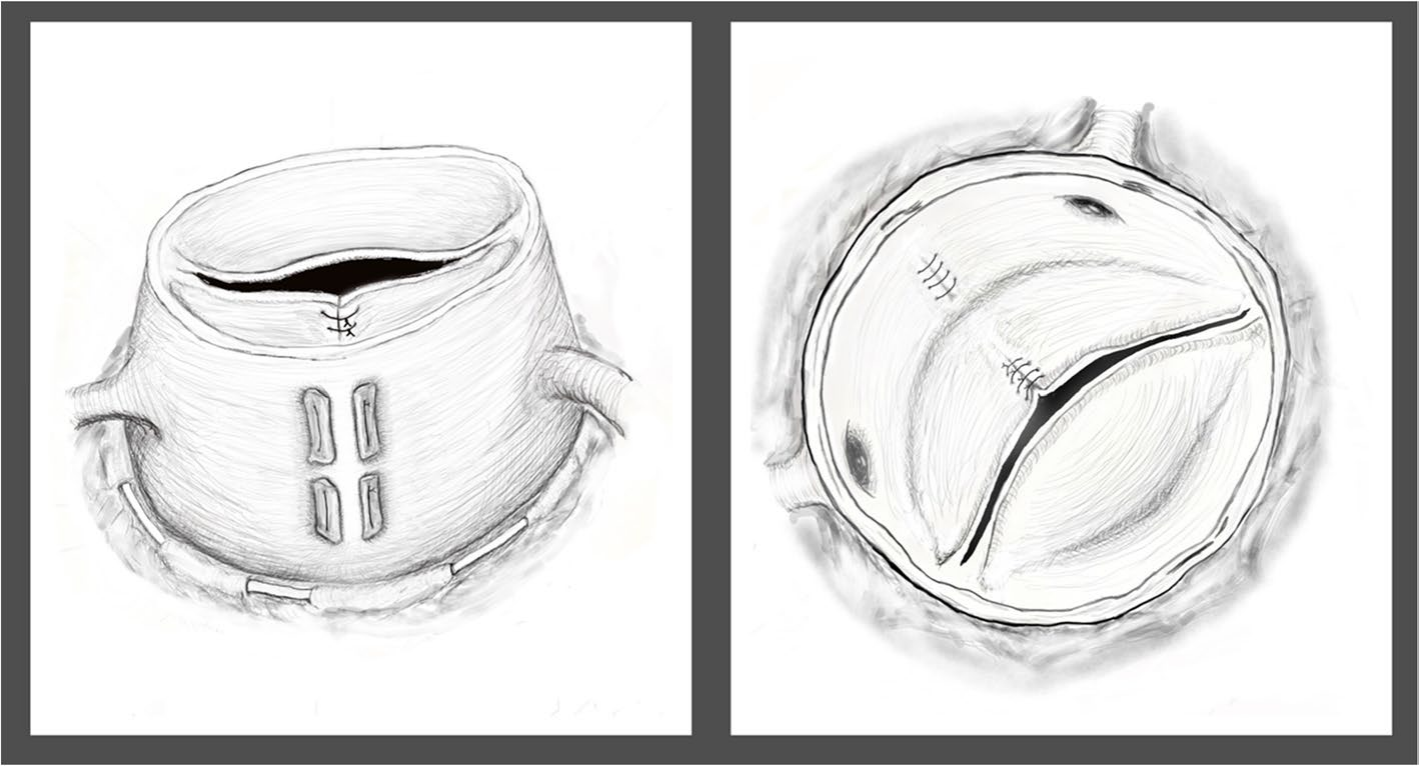
Two mattress sutures of 4–0 polypropylene, reinforced with autologous pericardium, were placed outside the fused cusp halfway from the basal ring to near the STJ

Following the Homburg group technique [9], the annuloplasty was performed using a 22-mm Hegar dilator and polytetrafluorethylene suture (Gore-Tex CV-0; W. L. Gore Associates, Munich, Germany). Triangular resection of the bulking raphe tissue with central cusp plication was necessary to create flexible coaptation and equalize the free margin lengths of both the fused and non-fused cusps. Sinus plication was added owing to the asymmetrical configuration of the Valsalva structure, where fused cusp area appeared larger than the other areas. Two mattress sutures of 4–0 polypropylene, reinforced with autologous pericardium, were placed outside the fused cusp from the basal ring to near the STJ, reducing the sinus circumference by approximately 15 mm (Fig. 3). Ascending aortic replacement was performed using a 22-mm InterGard prosthesis (Intervascular, La Ciotat, France). In the proximal suture, the aorta and prosthesis graft were carefully sutured to ensure the commissures were aligned on opposite sides. The repaired valve was inspected via endoscopy, while the graft was clamped, and crystalloid cardioplegia was injected to pressurize the aortic root. Based on the configurational information provided by endoscopy, central plication of both the fused and non-fused cusps was performed to achieve equal effective heights and good coaptation. After weaning from cardiopulmonary bypass, transesophageal echocardiography revealed trivial AR and modified commissural angulation. The operation, cardiopulmonary bypass, and aortic cross-clamping times were 311, 129, and 95 min, respectively.

**Fig. 4:**
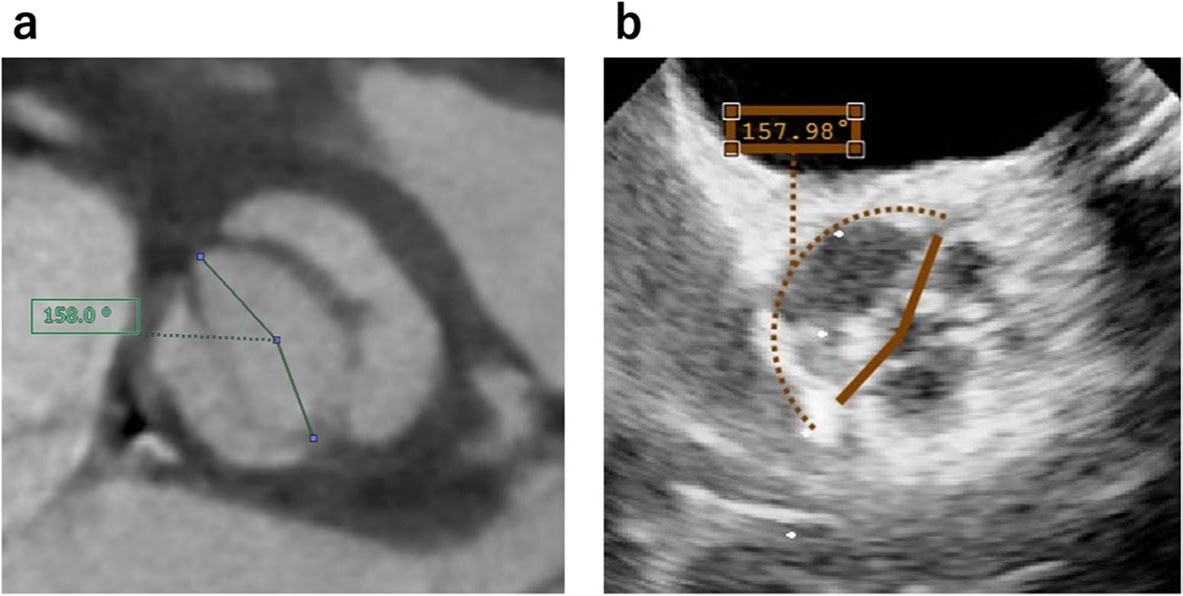
Postoperative contrast-enhanced computed tomography and transesophageal echocardiography demonstrating an increased commissural angle by approximately 20°

**Fig. 5:**
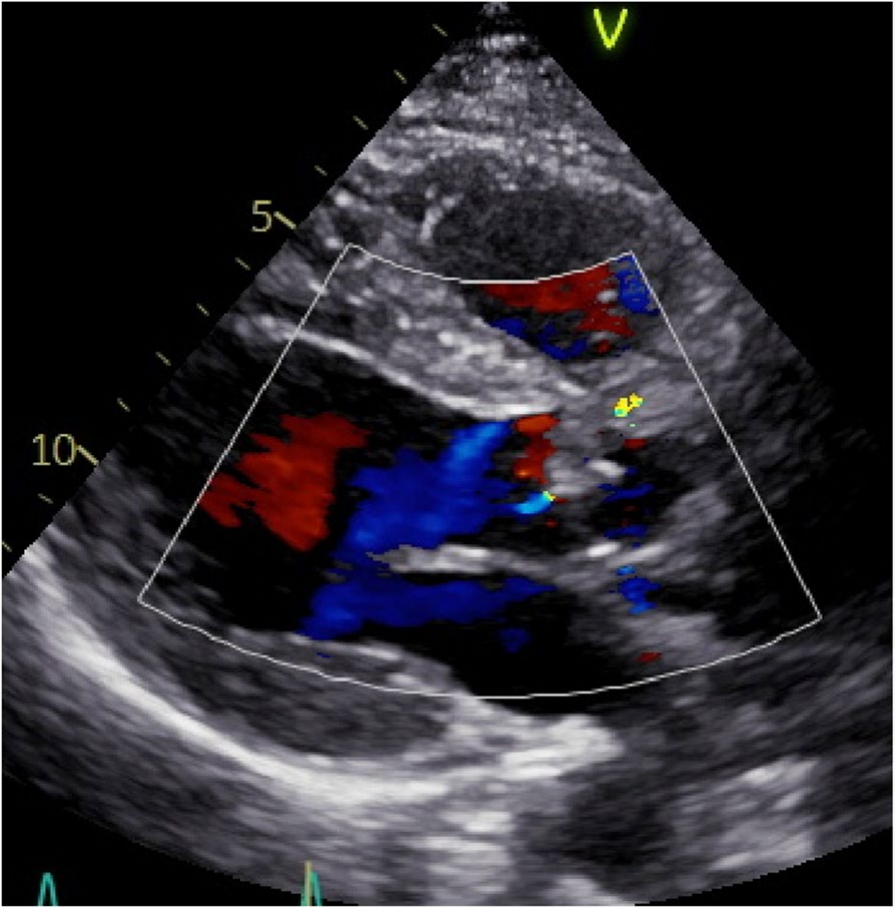
Postoperative echocardiography demonstrating trivial aortic regurgitation

The clinical course was uneventful, without major complications. Postoperative contrast-enhanced computed tomography and transesophageal echocardiography showed an improved commissural angle (Fig. 4). Postoperative contrast-enhanced computed tomography revealed an aortic root dimension of 23.6 mm at the aortic annulus, 33.0 mm at the sinus of Valsalva, and 20.0 mm at the STJ. Postoperative echocardiography revealed trivial AR (Fig. 5), and the mean transvalvular pressure gradient was 14.8 mmHg. At the 3-month follow-up, echocardiography demonstrated a substantial reduction in left ventricular volume and an improved left ventricular ejection fraction. The mean transvalvular pressure gradient further decreased to 11.1 mmHg (Table 1).

**Table 1:** Mean transvalvular pressure gradient

	**Preoperative**	**Postoperative**	**3-month follow-up**
LVEF (%)	50	47	54
LVEDV (ml)	259	231	113
LVESV (ml)	120	122	52
LVDd (mm)	72	66	53
LVDs (mm)	56	53	35
AV Vmax (m/s)	2.77	2.59	2.31
AV pPG (mmHg)	30.7	26.9	21.4
AV mPG (mmHg)	18	14.8	11.1
AR	Severe	Trivial	Trivial

*LVEF *left ventricular ejection fraction, *LVEDV *left ventricular end-diastolic volume, *LVESV *left ventricular end-systolic volume, *LVDd *left ventricular end-diastolic diameter, *LVDs *left ventricular end-systolic diameter, *AV *aortic valve, *Vmax *maximum blood flow velocity, *pPG *peak pressure gradient, *mPG *mean pressure gradient, *AR *aortic regurgitation

## Discussion

Reconstruction of regurgitant BAVs is a feasible alternative to aortic valve replacement because of its long-term durability [1, 3, 4]. Achieving proper annular size and symmetrical commissural orientation is essential for maintaining durability, as a dilatated AVD (≥ 29 mm) and asymmetrical commissural orientation (< 160°) are noteworthy predictors of recurrent AR [1]. Root replacement can address these issues, although most surgeons may aim to avoid this high-risk procedure.

Sinus plication is a valuable technique for maintaining repair durability without requiring root replacement, particularly in patients without relevant aortic root dilatation [6]. This procedure reduces the basal sinus circumference by approximately 15 mm and increases the angle of the non-fused cusp commissure by 20–30° [6]. A commissural orientation of > 160° results in nearly normal flow patterns in the ascending aorta [10]. Additionally, Arimura et al. reported that sinus plication reduced both the peak and mean transvalvular pressure gradients in experimental hydrodynamic models [7]. The average change in the commissural angle in their study was 10°, smaller than previously reported [6], possibly owing to the single sinus plication procedure. However, they hypothesized that plicating the sinus of Valsalva from the STJ to the annulus may enhance the effects of sinus plication on annuloplasty and effective height elevation [7].

In our case, we performed suture annuloplasty and ascending aortic replacement, alongside sinus plication. Postoperative TEE and CT showed commissural angle increased by around 20°, which was far from a symmetrical angle. Nevertheless, considering that the preoperative commissural angle was very asymmetrical (approximately 136°), the improved angle was compatible with the previous report [6].

The mean and peak transvalvular pressure gradients gradually decreased during follow-up. These findings were consistent with those reported by Arimura et al. [7]. Sinus plication may contribute to improvement of flow dynamics rather than structural elements. Consequently, continuous pressure on the repaired valves may be relieved, potentially preventing postoperative aortic stenosis.

## Conclusions

This report presents a case of successful aortic valve repair, including sinus plication, for a bicuspid aortic valve. Sinus plication may be one of the most effective aortic repair techniques for asymmetrical bicuspid aortic valves. It is particularly useful in the absence of a Valsalva sinus aneurysm to avoid postoperative aortic valve stenosis.

## Data Availability

The datasets used during the current study are available from the corresponding author on reasonable request.

## Abbreviations

BAV: Bicuspid aortic valve
AR: Aortic regurgitation
STJ: Sinotubular junction
AVD: Aortoventricular diameter
